# Missouri Valley Veterinary Medical Association

**Published:** 1897-08

**Authors:** E. P. Schaffter

**Affiliations:** Secretary pro tem.


### MISSOURI VALLEY VETERINARY MEDICAL ASSOCIATION.

THE third annual meeting was held in Kansas City, Mo., July 9, 1897. The meeting was called to order by the President, Dr. Stewart. The Secretary, Dr. Hunter, being absent, Dr. Schaffter was elected Secretary *pro tem.* The first order of business was roll-call, the following members responding: Drs. Stewart, Moore, Bray, and Schaffter. Members of the profession present were Drs. Heck, Steddom, and Johnson. The minutes of the February meeting were read and approved. Correspondence between the Secretary and Dr. T. J. Turner was read, which requested his release from the Association on account of his inability to attend meetings, being located at Indianapolis. Upon motion, duly seconded, Dr. Turner's resignation was accepted. Applications for membership were made by Drs. Heck, Steddom, and Johnson. The Board of Censors reported favorably upon the petitions, and upon motion, duly seconded, they were declared members. The President was authorized to appoint delegates to the U. S. V. M. A. meeting, which will be held at Nashville, Tenn.

A paper was read by Dr. Moore: "Sub-puncturing for Gastric Flatulence." The subject elicited a full discussion by all present. Dr. Bray volunteered a little talk on uses and abuses of actual cautery. The discussion following brought out many interesting and valuable points.

Election of officers resulted as follows: Dr. Hunter, President; Dr. Moore, Vice-President; Dr. McCurdy, Second Vice-President; Dr. Schaffter, Secretary and Treasurer; Censors, Drs. Bray, Heck, Steddom, Johnson, and Harrison. Meeting adjourned to meet with October regular meeting.

E. P. SCHAFFTER, V.S.,
Secretary pro tem.

